# New Books

**Published:** 2007-05

**Advances in Data Analysis**

Reinhold Decker, Hans-Joachim Lenz, eds.

New York:Springer, 2007. 687 pp. ISBN: 3-540-70980-0, $169

**Agricultural Water Management**

Laura Holliday, ed.

Washington, DC:National Academies Press, 2007. 158 pp. ISBN: 0-309-10603-6, $34.50

**Arsenic in Soil and Groundwater Environment**

Prosun Bhattacharya, Arun B. Mukherjee, Jochen Bundschuh, Ron Zevenhoven, Richard Loeppert, eds.

Burlington, MA:Elsevier, 2007. 670 pp. ISBN: 0-444-51820-7, $120

**Bioavailability, Bioaccessibility and Mobility of Environmental Contaminants**

John R. Dean

Hoboken, NJ:John Wiley & Sons, Inc., 2007. 316 pp. ISBN: 0-470-02577-2, $155

**Cancer**

Rob Bradbury, ed.

New York:Springer, 2007. 451 pp. ISBN: 3-540-33119-3, $149

**Cytokines in Human Health**

Robert V. House, Jacques Descotes

Totowa, NJ:Humana Press, 2007. 448 pp. ISBN: 1-58829-467-6, $150

**Elements of Environmental Chemistry**

Ronald A. Hites

Hoboken, NJ:John Wiley & Sons, Inc., 2007. 224 pp. ISBN: 0-471-99815-0, $39.95

**Energy and American Society—Thirteen Myths**

Benjamin K. Sovacool, Marilyn A. Brown, eds.

New York:Springer, 2007. 371 pp. ISBN: 1-4020-5563-8, $79.95

**Epigenetics**

C. David Allis, Thomas Jenuwein, Danny Reinberg, Marie-Laure Caparros, eds.

Woodbury, NY:Cold Spring Harbor Laboratory Press, 2007. 502 pp. ISBN: 0-8796-9724-2, $150

**Gene Transfer: Delivery and Expression of DNA and RNA**

Theodore Friedmann, John Rossi, eds.

Woodbury, NY:Cold Spring Harbor Laboratory Press, 2007. 793 pp. ISBN: 0-8796-9765-5, $159

**Global Environmental Governance**

James Gustave Speth, Peter M. Haas

Washington, DC:Island Press, 2006. 179 pp. ISBN: 1-59726-081-9, $19.95

**Life Science Data Mining**

Stephen Wong, Chung-Sheng Li, eds.

Hackensack, NJ:World Scientific Publishing Co., 2007. 388 pp. ISBN: 981-270-064-3, $84

**Planet Earth**

Alastair Fothergil

Berkeley:University of California Press, 2007. 312 pp. ISBN: 0-520-25054-3, $39.95

**Radioactivity in the Terrestrial Environment**

G. Shaw, ed.

Burlington, MA:Elsevier, 2007. 306 pp. ISBN: 0-08-043872-5, $145

**Recombinant DNA: Genes and Genomes—A Short Course, 3rd ed.**

James D. Watson, Amy A. Caudy, Richard M. Myers, Jan A. Witkowski

Woodbury, NY:Cold Spring Harbor Laboratory Press, 2007. 474 pp. ISBN: 0-7167-2866-5, $92.30

**Regulatory RNAs**

Bruce Stillman, David Stewart, eds.

Woodbury, NY:Cold Spring Harbor Laboratory Press, 2007. 574 pp. ISBN: 0-8796-9817-1, $295

**The Cigarette Century: The Rise, Fall, and Deadly Persistance of the Product that Defined America**

Allan Brandt

New York:Basic Books, 2007. 600 pp. ISBN: 0-465-07047-7, $36

**The First Scientific American: Benjamin Franklin and the Pursuit of Genius**

Joyce Chaplin

New York:Basic Books, 2007. 352 pp. ISBN: 0-465-00956-5, $17.50

**The Most Important Fish in The Sea: Menhaden in America**

H. Bruce Franklin

Washington, DC:Island Press, 2007. 280 pp. ISBN: 1-59726-164-5, $25

**The Revenge of Gaia: Earth’s Climate Crisis and The Fate of Humanity**

James Lovelock

New York:Basic Books, 2007. 192 pp. ISBN: 0-465-04169-8, $15.95

**This Moment on Earth: Today’s New Environmentalists and Their Vision for the Future**

John Kerry, Teresa Heinz Kerry

Jackson, TN:Public Affairs, 2007. 240 pp. ISBN: 1-5864-8431-1, $25

